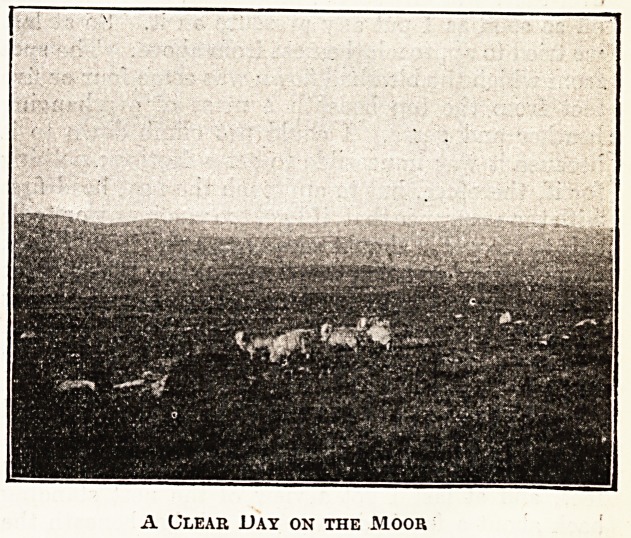# The Haunt of the Ring Ouzel

**Published:** 1913-04-12

**Authors:** 


### RELAXATIONS AND HOBBIES.

### The Haunt of the Ring Ouzel.

It has been said of the Ring Ouzel, or, as he is locally called, the Mountain Blackbird, that his song is somewhat monotonous and derives its principal charm from the surroundings in which it is heard. The same may be said with equal truth of the songs and call-notes of the majority of birds. There is nothing intrinsically attractive about the barking croak of a raven, but among the steep rocks towering above a lonely tarn in the Welsh mountains one cannot hear it without a thrill of pure pleasure. The

The Site of the First Ring Ouzel's Nest.

cry of a curlew on the moors, the ceaseless chatter of a sedge warbler by a chalk-stream, and, again, the crooning of a turtle-dove in a peaceful, secluded thicket delight one less because they have any special musical qualities than because they fit, or, through long association, seem to fit so admirably with the surroundings in which they are heard.

So it is with most of the sounds of Nature. The pleasure of hearing what one expects to hear and is listening for brings into play an additional sense, focussing, so to speak, the enjoyment of all the other senses, and justifying expectation based on past experience. I have always been inclined to regard the ring ouzel as the exception to the general rule that birds fit the localities in which they are found. I used to see him looking curiously lonely and out of place always on the high moor, where bird life, if one excepts the innumerable meadow pipits, is most scarce, and his song seemed to be the expression of his loneliness. No other thrush (I use the word in the generic sense) ever came within earshot of these solitudes, and this brownish-black bird, with his white breast-band and the habit of a common blackbird, seemed to be the victim of some social ostracism which he tried to carry off with a bold front. The great simplicity of his song, less finished and less confident than that of the song-thrush or blackbird, was exaggerated by the breadth of his surroundings and by the fact that the bird was always solitary. It is, nevertheless, true that the song derives its charm from the surroundings in which it is heard. In the chorus by the side of a coppice in the Midlands or by a chalk-stream in Wiltshire or Hampshire it would hardly attract attention at all; in its own place it cannot fail to be noticed, and, I think, to charm.

My acquaintance with the ring ouzel during the mating and breeding season is confined to one locality—the high levels of Dartmoor—and although I became familiar some years since with the appearance and song of the bird in early spring—that is to say, shortly after its arrival from the South—it was only comparatively recently that I was able, for the first time, to visit Dartmoor late enough in the year to entertain any serious hope of finding its nest.

When at last, after an interval of some years, during which I had not been able to visit Dartmoor at all, I was able to realise my hope of spending a short holiday there in May 1910 I suffered a temporary disappointment. I do not refer to the weather, which was abominable. I was living under canvas, which made it seem worse; but I have learnt to expect the worst of this moody month. My disappointment sprang from another cause than its vagaries. I lost no time in inspecting the places where in former days I had found ring ouzels in comparative abundance, and they were not there. A good deal of my time was devoted to other birds, but I was always on the look out for the ring ouzel and, although I wandered over miles of moor I failed, for more than a week, to find a single bird of the species I was, for the time being, chiefly concerned with. In short, I learned that the ring

A Clear Day on the Moor ouzel, when he is singing for a mate, as he is in April, was a far easier bird to find than the ring ouzel engaged upon his family affairs. Gradually my search drew me further and further up into the highest and most solitary parts of the moor. I had made careful inquiries as to the most likely spots in which to find a nest, and had been told that, on the whole, the steep rocky sides of old quarry cuttings were the most favoured spots, but that the nests were not infrequently found in the banks by the high roads. On the latter point I felt some scepticism, which may, for all I know, have been unfounded. At any rate, I directed my attention chiefly to the more remote parts of the moor, and especially to every gully I could espy.

I can quite understand that to many people it must seem mere foolishness to spend whole days in hunting for the nest of a comparatively insignificant bird, and, if that were all, I should be prepared to agree. But I hold that a bird is only half known till you have followed it to the nest, and I confess without shame that I was not more excited over my first blackbird's nest as a boy (and well I remember it) than over the ring ouzel's nest when at last it appeared to be within the region of discovery. The sun was already low when we chanced upon a deep gully which we had not hitherto explored. It was so late, indeed, and we were so far from home that I had some doubt whether it might not be better to leave it till another day. But my sister, who was the companion of my search and had braved the rigours of a May camp to make acquaintance with the birds of Dartmoor, elected to search it at once. She walked along the top of one side and I stumbled along over the rough ground in the middle track. About half-way along we both gave vent to a sort of view holloa. A rusty-coloured bird had slipped out of the rocks on the side opposite to that on which my sister walked. There could be no doubt about the proximity of the nest. As it happened, my sister's keen eye had marked the very spot from which the bird came. There followed, for me, a palpitating struggle. Below the nest was sheer rock for some twenty to thirty feet. I tried to climb it, but, wherever there was any sort of projection, the rock scaled off as soon as I put any pressure on it. So at last we tried to approach the nest from above. The spot from which the bird had flown was some four or five feet from the top beneath a mass of overhanging heather and gorse. I could not climb down to it because it was impossible to get a footing; nothing for it, therefore, but to approach the nest head first with the assurance that, if I got too much way on to be able to recover, I should take a header on to solid rock about thirty feet below me. I did not like it at all, but it was impossible to turn back when the prize was obviously so near, and to complete my assurance on this point, the bird returned to a spot just opposite the nest and, for the first time in my life, I heard the alarm call of the ring ouzel, a loud-repeated "teck, teck." So I hung on to the heather with my hands, and my sister clung desperately to my feet, and at last I got a view of the nest standing back about a foot's distance on a ledge beneath the overhanging heather and gorse, and, gingerly letting go with one hand, I successfully inserted it in the nest and abstracted an egg. With the egg in my mouth I wriggled back to security.

Amateur egg-collecting is, I think, difficult to defend, but the collecting instinct is curiously strong, and the ring ouzel's egg was designed to fill a gap in a collection dating from my early boyhood, which consists entirely of eggs found and identified by myself and one of my brothers. It has been made in a limited area and on fixed principles. In the first place any egg placed in it must have been found and its authenticity carefully verified by one of ourselves. In the next not more than one, or at most two, eggs of a species are taken from the same nest. Thirdly, eggs of each species are added from other nests as opportunity offers till the number in the collection is that of the average clutch, care being exercised to secure specimens as far as possible with different characteristics. Fourthly, after the number of the average clutch has been reached no further additions of the particular species are made except to replace specimens accidentally damaged, or because a specimen is found presenting peculiarities of size, colour, or marking. I think it must be admitted that a collection made on these principles possesses a certain degree of scientific interest and involves the least degree of cruelty and destruction of bird life. I have nothing but contempt for amateur collections of bought rarities—in fact I should like to make the sale and purchase of birds' eggs to and by any except recognised scientific societies a penal offence, and I should like to see the law properly enforced. By this digression on the subject of my own egg collection I have shown that I think any amateur collection needs an apology; but having made my apology I admit without hesitation that we gloated over this new egg—barely distinguishable from a common blackbird's egg—as over a pearl of great price. There was only one element of dissatisfaction about the find. It was absolutely impossible to take a photograph of the nest. We decided, however, to return on the next or the following day with the camera to photograph the spot and, having arrived at this decision, moved off to make way for the, by now, highly-agitated bird.

The next day was stormy, but the day following was not only fine but gloriously fine, and we trudged right up the moor with the camera to the gully of discovery and photographed the situation of the nest. The charm of the surroundings of the ring ouzel was never so strongly presented to my mind. It was the day on which the earth was, according to the scientists, passing through the tail of Halley's comet, and I imagine, especially as I received reports of similar atmospheric effects from other parts of the country, that the extraordinary brightness and clearness of the atmosphere was due in some degree to this circumstance. At brief intervals throughout our walk our footsteps were arrested by some new aspect of the glorious panorama spread out before us and, as we sat at the head of the gully to eat our luncheon, we could literally count the hedges of the fields in the low ground beyond the moor in the direction of Paignton and Torquay right away to the sea. We could distinguish the very stones of the quay in what we took to be Paignton, and the ships beyond were clearly visible. A photograph which I took with a Kodak camera of a typical Dartmoor scene shows, by its marvellous distances, the unusual absence of any obscuring haze.

*(To be continued.)*

## Figures and Tables

**Figure f1:**
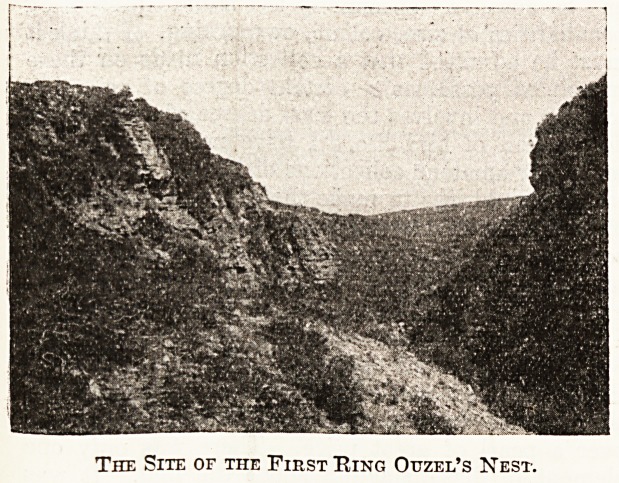


**Figure f2:**